# 5-Methyl-3-(3-methyl­phen­yl)-7-phenyl-1,2,4-triazolo[4,3-*c*]pyrimidine

**DOI:** 10.1107/S1600536811010683

**Published:** 2011-03-26

**Authors:** Jasmin Preis, Dieter Schollmeyer, Heiner Detert

**Affiliations:** a University Mainz, Duesbergweg 10-14, 55099 Mainz, Germany

## Abstract

The title compound, C_19_H_16_N_4_, is one of the few known 3,7-diaryl-1,2,4-triazolo[4,3-*c*]pyrimidines. The triazolopyrimidine unit is essentially planar (r.m.s. deviation = 0.048 Å). The phenyl ring and the heterocyclic core subtend a dihedral angle of only 15.09 (6)°, whereas the *m*-tolyl ring is twisted by 71.80 (6)° out of the plane of the triazole ring. Two C—H⋯N hydrogen bonds and π–π stacking inter­actions [centroid–centroid distance = 3.7045 (8) Å] stabilize the crystal packing.

## Related literature

For the synthesis of higher conjugated and annulated heterocyclic π-systems, see: Detert & Schollmeyer (1999 ▶); Sugiono & Detert (2001 ▶). The acyl­ation of tetra­zoles with chloro­azines and thermal ring transformation leads to triazolo annulated azines, see: Huisgen, Sauer & Seidel (1960 ▶); Huisgen, Sturm & Markgraf (1960 ▶); Huisgen *et al.* (1961 ▶); Glang *et al.* (2008 ▶). Whereas a broad variety of triazolopyrimidines are known, only two further [1,2,4]triazolo[4,3-*c*]pyrimidines with a 3,7-diaryl substitution have been reported so far, see: Seada *et al.* (1992 ▶).
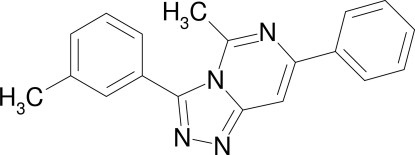


## Experimental

### 

#### Crystal data

C_19_H_16_N_4_
*M* *_r_* = 300.36
Triclinic, 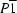

*a* = 6.4270 (4) Å
*b* = 11.1706 (6) Å
*c* = 11.3672 (7) Å
α = 79.963 (5)°
β = 74.894 (5)°
γ = 81.877 (5)°
*V* = 771.88 (8) Å^3^
*Z* = 2
Cu *K*α radiation
μ = 0.63 mm^−1^
*T* = 193 K
0.45 × 0.40 × 0.25 mm

#### Data collection

Enraf–Nonius CAD-4 diffractometer
3207 measured reflections
2924 independent reflections
2573 reflections with *I* > 2σ(*I*)
*R*_int_ = 0.088
3 standard reflections every 60 min  intensity decay: 2%

#### Refinement

*R*[*F* ^2^ > 2σ(*F* ^2^)] = 0.042
*wR*(*F* ^2^) = 0.119
*S* = 1.03
2924 reflections
211 parameters
H-atom parameters constrained
Δρ_max_ = 0.25 e Å^−3^
Δρ_min_ = −0.21 e Å^−3^

### 

Data collection: *CAD-4 Software* (Enraf–Nonius, 1989 ▶); cell refinement: *CAD-4 Software*; data reduction: *CORINC* (Dräger & Gattow, 1971 ▶); program(s) used to solve structure: *SIR97* (Altomare *et al.*, 1999 ▶); program(s) used to refine structure: *SHELXL97* (Sheldrick, 2008 ▶); molecular graphics: *PLATON* (Spek, 2009 ▶); software used to prepare material for publication: *PLATON*.

## Supplementary Material

Crystal structure: contains datablocks I, global. DOI: 10.1107/S1600536811010683/bt5497sup1.cif

Structure factors: contains datablocks I. DOI: 10.1107/S1600536811010683/bt5497Isup2.hkl

Additional supplementary materials:  crystallographic information; 3D view; checkCIF report

## Figures and Tables

**Table 1:** Hydrogen-bond geometry (Å, °)

*D*—H⋯*A*	*D*—H	H⋯*A*	*D*⋯*A*	*D*—H⋯*A*
C6—H6⋯N8^i^	0.95	2.53	3.4597 (17)	166
C23—H23⋯N8^i^	0.95	2.61	3.5374 (19)	167

Symmetry code: (i) 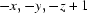.

**Fig. 1.**
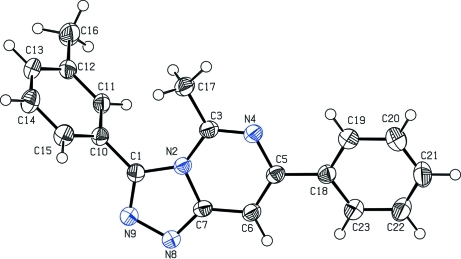
View of the title compound. Displacement ellipsoids are drawn at the 50% probability level.

**Fig. 2.**
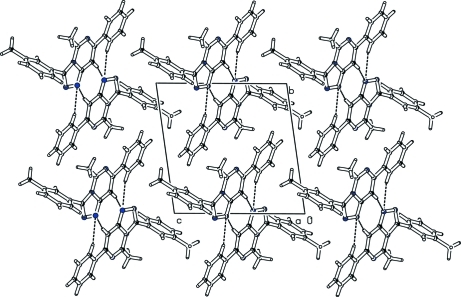
Part of the packing diagram showing the hydrogen bonds and the π-π-interactions. View along a axis.

## supplementary crystallographic information

### Comment

The title compound was synthesized as part of a larger project focusing on the synthesis of higher conjugated and annulated heterocyclic π-systems see Detert & Schollmeyer (1999), Sugiono & Detert (2001). The acylation of tetrazoles followed by thermal ring transformation is a highly efficient route for the synthesis of 1,3,4-oxadiazoles and triazoles (Huisgen, Sauer & Seidel 1960; Huisgen,   Sturm  & Markgraf, 1960) and can also be applied to 2-chloroazines to yield triazolo-annulated azines. In the crystals of the title compound, the phenyl ring is only slightly turned out of the plane of the heterocyclic core [dihedral angle of 15.09 (6)°], the angle between the mean planes of the core and the *m*-tolyl ring amounts to 71.80 (6)°. Two molecules of the title compound form a dimer connected *via* hydrogen bonds C6—H6···N8 (2.53 Å) and C23—H23···N8 (2.61 Å). In the crystal, the dimers are connected *via*π-π-interactions between the rings with a distance of the triazoles (C1—N2, C7—N9) of 3.5404 (8) Å and of the pyrimidines (N2—C7) of 3.7045 (8) Å.

### Experimental

The title compound was prepared by adding 2,4,6-collidine (0.54 g, 4.5 mmol) to a solution of 4-chloro-2-methyl-6-phenylpyrimidine (0.61 g, 3 mmol) and 5-(3-methyl- phenyl)tetrazole in xylenes (60 ml) and heating until gas was evolved (363 K). Stirring and heating was continued for 6 h, the solvent removed *in vacuo* and the residue purified by chromatography (SiO_2_ /toluene/ethyl acetate = 1 / 1, *R*_f_ = 0.28). The title compound was isolated as a yellowish powder with m.p. = 412–413 K. Crystals were obtained by slow evaporation of a solution of the title compound in chloroform/hexanes. All spectrocopic data were in accordance with the assumed structure, but an unique proton-proton coupling over 6 bonds from the pyrimidine-H across the heterocycle to the methyl group was observed.

### Refinement

Hydrogen atoms   were placed at calculated positions with C—H = 0.95 Å (aromatic) or 0.98–0.99 Å (*sp*^3^ C-atom). All H atoms were refined in the riding-model approximation with isotropic displacement parameters  set at 1.2–1.5 times of the *U*_eq_ of the parent atom.

### Crystal data

**Table:**

C_19_H_16_N_4_	*Z* = 2
*M**_r_* = 300.36	*F*(000) = 316
Triclinic, *P*1	*D*_x_ = 1.292 Mg m^−^^3^
Hall symbol: -P 1	Melting point: 412 K
*a* = 6.4270 (4) Å	Cu *K*α radiation, λ = 1.54178 Å
*b* = 11.1706 (6) Å	Cell parameters from 25 reflections
*c* = 11.3672 (7) Å	θ = 61–70°
α = 79.963 (5)°	µ = 0.63 mm^−^^1^
β = 74.894 (5)°	*T* = 193 K
γ = 81.877 (5)°	Plate, yellow
*V* = 771.88 (8) Å^3^	0.45 × 0.40 × 0.25 mm

### Data collection

**Table:**

Enraf–Nonius CAD-4 diffractometer	*R*_int_ = 0.088
Radiation source: rotating anode	θ_max_ = 69.9°, θ_min_ = 4.0°
graphite	*h* = −7→0
ω/2θ scans	*k* = −13→13
3207 measured reflections	*l* = −13→13
2924 independent reflections	3 standard reflections every 60 min
2573 reflections with *I* > 2σ(*I*)	intensity decay: 2%

### Refinement

**Table:**

Refinement on *F*^2^	Secondary atom site location: difference Fourier map
Least-squares matrix: full	Hydrogen site location: inferred from neighbouring sites
*R*[*F*^2^ > 2σ(*F*^2^)] = 0.042	H-atom parameters constrained
*wR*(*F*^2^) = 0.119	*w* = 1/[σ^2^(*F*_o_^2^) + (0.0657*P*)^2^ + 0.1681*P*] where *P* = (*F*_o_^2^ + 2*F*_c_^2^)/3
*S* = 1.03	(Δ/σ)_max_ < 0.001
2924 reflections	Δρ_max_ = 0.25 e Å^−^^3^
211 parameters	Δρ_min_ = −0.21 e Å^−^^3^
0 restraints	Extinction correction: *SHELXL97* (Sheldrick, 2008), Fc^*^=kFc[1+0.001xFc^2^λ^3^/sin(2θ)]^-1/4^
Primary atom site location: structure-invariant direct methods	Extinction coefficient: 0.0261 (19)

### Special details

**Table:**

Experimental. ^1^H-NMR (CDCl_3_): 8.06 (d, 1 H), 8.03 (d 1 H), 7.90 (s, 1H, H-5 pyrimidin), 7.45 (m, 7 H), 2.47 (S, 3 H, CH_3_), 2.43 (3, 3 H, CH_3_); ^13^C-NMR (CDCl_3_): 151.4, 149.2, 147.1, 146.3, 138.3, 136.1, 131.5, 131.4, 129.9, 128.9, 128.2, 128.0, 127.9, 103.0, 23.6, 21.4; MS (FD): 300.1 (100%, M^+^), 600.3 (8% M_2_^+^), 900.3 (M_3_^+^). UV-vis: λ_max _=393nm (CH_2_Cl_2_), fluorescence: λ_max _=503nm (CH_2_Cl_2_).
Geometry. All e.s.d.'s (except the e.s.d. in the dihedral angle between two l.s. planes) are estimated using the full covariance matrix. The cell e.s.d.'s are taken into account individually in the estimation of e.s.d.'s in distances, angles and torsion angles; correlations between e.s.d.'s in cell parameters are only used when they are defined by crystal symmetry. An approximate (isotropic) treatment of cell e.s.d.'s is used for estimating e.s.d.'s involving l.s. planes.
Refinement. Refinement of *F*^2^ against ALL reflections. The weighted *R*-factor *wR* and goodness of fit *S* are based on *F*^2^, conventional *R*-factors *R* are based on *F*, with *F* set to zero for negative *F*^2^. The threshold expression of *F*^2^ > σ(*F*^2^) is used only for calculating *R*-factors(gt) *etc*. and is not relevant to the choice of reflections for refinement. *R*-factors based on *F*^2^ are statistically about twice as large as those based on *F*, and *R*- factors based on ALL data will be even larger.

### Fractional atomic coordinates and isotropic or equivalent isotropic displacement parameters (Å^2^)

**Table:**

	*x*	*y*	*z*	*U*_iso_*/*U*_eq_	
C1	0.4291 (2)	0.07105 (12)	0.69733 (11)	0.0279 (3)	
N2	0.35695 (17)	0.15861 (10)	0.60994 (9)	0.0269 (3)	
C3	0.3949 (2)	0.27979 (12)	0.56152 (12)	0.0295 (3)	
N4	0.30230 (19)	0.33877 (10)	0.47760 (10)	0.0322 (3)	
C5	0.1646 (2)	0.28265 (12)	0.43301 (12)	0.0291 (3)	
C6	0.1357 (2)	0.16223 (12)	0.46841 (12)	0.0300 (3)	
H6	0.0468	0.1239	0.4343	0.036*	
C7	0.2419 (2)	0.09577 (12)	0.55754 (12)	0.0275 (3)	
N8	0.24802 (18)	−0.02029 (10)	0.60599 (10)	0.0317 (3)	
N9	0.36567 (18)	−0.03428 (10)	0.69480 (10)	0.0318 (3)	
C10	0.5391 (2)	0.09494 (12)	0.78900 (12)	0.0284 (3)	
C11	0.4254 (2)	0.16174 (12)	0.88241 (12)	0.0315 (3)	
H11	0.2792	0.1931	0.8849	0.038*	
C12	0.5209 (2)	0.18358 (12)	0.97209 (12)	0.0334 (3)	
C13	0.7358 (2)	0.13693 (13)	0.96559 (13)	0.0371 (3)	
H13	0.8051	0.1516	1.0252	0.045*	
C14	0.8494 (2)	0.06977 (15)	0.87386 (14)	0.0404 (4)	
H14	0.9956	0.0384	0.8713	0.049*	
C15	0.7522 (2)	0.04762 (14)	0.78542 (13)	0.0355 (3)	
H15	0.8305	0.0006	0.7230	0.043*	
C16	0.3970 (3)	0.25436 (16)	1.07371 (15)	0.0502 (4)	
H16A	0.4322	0.3392	1.0529	0.075*	
H16B	0.4366	0.2176	1.1507	0.075*	
H16C	0.2413	0.2525	1.0838	0.075*	
C17	0.5494 (3)	0.33769 (13)	0.60606 (13)	0.0379 (3)	
H17A	0.5796	0.4161	0.5543	0.057*	
H17B	0.6845	0.2838	0.6018	0.057*	
H17C	0.4855	0.3513	0.6915	0.057*	
C18	0.0596 (2)	0.36189 (12)	0.34111 (12)	0.0316 (3)	
C19	0.1311 (3)	0.47490 (14)	0.28749 (15)	0.0450 (4)	
H19	0.2440	0.5028	0.3121	0.054*	
C20	0.0393 (3)	0.54751 (16)	0.19826 (17)	0.0555 (5)	
H20	0.0908	0.6243	0.1615	0.067*	
C21	−0.1258 (3)	0.50869 (16)	0.16287 (16)	0.0522 (4)	
H21	−0.1885	0.5584	0.1017	0.063*	
C22	−0.2000 (3)	0.39726 (16)	0.21656 (16)	0.0476 (4)	
H22	−0.3149	0.3707	0.1926	0.057*	
C23	−0.1088 (2)	0.32384 (14)	0.30495 (14)	0.0383 (3)	
H23	−0.1610	0.2472	0.3412	0.046*	

### Atomic displacement parameters (Å^2^)

**Table:**

	*U*^11^	*U*^22^	*U*^33^	*U*^12^	*U*^13^	*U*^23^
C1	0.0275 (6)	0.0296 (6)	0.0261 (6)	−0.0006 (5)	−0.0059 (5)	−0.0052 (5)
N2	0.0276 (5)	0.0283 (6)	0.0264 (5)	−0.0024 (4)	−0.0077 (4)	−0.0067 (4)
C3	0.0327 (7)	0.0293 (7)	0.0279 (6)	−0.0057 (5)	−0.0075 (5)	−0.0054 (5)
N4	0.0381 (6)	0.0315 (6)	0.0301 (6)	−0.0064 (5)	−0.0125 (5)	−0.0040 (5)
C5	0.0292 (6)	0.0320 (7)	0.0275 (6)	−0.0024 (5)	−0.0072 (5)	−0.0084 (5)
C6	0.0306 (7)	0.0320 (7)	0.0307 (7)	−0.0019 (5)	−0.0106 (5)	−0.0096 (5)
C7	0.0264 (6)	0.0292 (6)	0.0290 (6)	−0.0031 (5)	−0.0064 (5)	−0.0099 (5)
N8	0.0349 (6)	0.0293 (6)	0.0344 (6)	−0.0021 (4)	−0.0137 (5)	−0.0067 (5)
N9	0.0337 (6)	0.0311 (6)	0.0329 (6)	−0.0014 (4)	−0.0121 (5)	−0.0059 (5)
C10	0.0305 (7)	0.0288 (7)	0.0265 (6)	−0.0035 (5)	−0.0088 (5)	−0.0022 (5)
C11	0.0319 (7)	0.0317 (7)	0.0317 (7)	0.0031 (5)	−0.0124 (5)	−0.0046 (5)
C12	0.0431 (8)	0.0293 (7)	0.0297 (7)	0.0017 (6)	−0.0144 (6)	−0.0046 (5)
C13	0.0426 (8)	0.0393 (8)	0.0344 (7)	−0.0020 (6)	−0.0209 (6)	−0.0029 (6)
C14	0.0307 (7)	0.0510 (9)	0.0410 (8)	0.0011 (6)	−0.0143 (6)	−0.0061 (7)
C15	0.0311 (7)	0.0423 (8)	0.0320 (7)	0.0010 (6)	−0.0062 (5)	−0.0088 (6)
C16	0.0641 (11)	0.0504 (9)	0.0417 (9)	0.0153 (8)	−0.0249 (8)	−0.0206 (7)
C17	0.0474 (8)	0.0352 (7)	0.0368 (7)	−0.0144 (6)	−0.0187 (6)	0.0008 (6)
C18	0.0355 (7)	0.0321 (7)	0.0285 (7)	0.0013 (5)	−0.0104 (5)	−0.0081 (5)
C19	0.0572 (10)	0.0350 (8)	0.0494 (9)	−0.0071 (7)	−0.0261 (8)	−0.0021 (7)
C20	0.0750 (12)	0.0369 (9)	0.0590 (11)	−0.0055 (8)	−0.0320 (9)	0.0051 (8)
C21	0.0667 (11)	0.0456 (9)	0.0472 (9)	0.0101 (8)	−0.0299 (8)	−0.0028 (7)
C22	0.0493 (9)	0.0526 (10)	0.0486 (9)	0.0026 (7)	−0.0275 (7)	−0.0101 (7)
C23	0.0395 (8)	0.0395 (8)	0.0395 (8)	−0.0025 (6)	−0.0162 (6)	−0.0065 (6)

### Geometric parameters (Å, °)

**Table:**

C1—N9	1.3057 (17)	C18—C23	1.3939 (19)
C1—N2	1.3899 (16)	C19—C20	1.387 (2)
C1—C10	1.4802 (17)	C20—C21	1.375 (2)
N2—C7	1.3846 (15)	C21—C22	1.379 (3)
N2—C3	1.3995 (17)	C22—C23	1.382 (2)
C3—N4	1.2901 (17)	C6—H6	0.9500
C3—C17	1.4890 (18)	C11—H11	0.9500
N4—C5	1.3909 (16)	C13—H13	0.9500
C5—C6	1.3598 (19)	C14—H14	0.9500
C5—C18	1.4854 (18)	C15—H15	0.9500
C6—C7	1.4136 (18)	C16—H16A	0.9800
C7—N8	1.3167 (17)	C16—H16B	0.9800
N8—N9	1.3873 (15)	C16—H16C	0.9800
C10—C15	1.3896 (19)	C17—H17A	0.9800
C10—C11	1.3914 (18)	C17—H17B	0.9800
C11—C12	1.3894 (18)	C17—H17C	0.9800
C12—C13	1.393 (2)	C19—H19	0.9500
C12—C16	1.502 (2)	C20—H20	0.9500
C13—C14	1.380 (2)	C21—H21	0.9500
C14—C15	1.385 (2)	C22—H22	0.9500
C18—C19	1.388 (2)	C23—H23	0.9500
			
N9—C1—N2	109.46 (11)	C21—C22—C23	120.60 (15)
N9—C1—C10	124.55 (12)	C22—C23—C18	120.25 (14)
N2—C1—C10	125.66 (11)	C5—C6—H6	121.00
C7—N2—C1	104.30 (10)	C7—C6—H6	121.00
C7—N2—C3	120.16 (11)	C10—C11—H11	119.00
C1—N2—C3	135.10 (11)	C12—C11—H11	119.00
N4—C3—N2	120.52 (12)	C12—C13—H13	119.00
N4—C3—C17	120.83 (12)	C14—C13—H13	120.00
N2—C3—C17	118.63 (11)	C13—C14—H14	120.00
C3—N4—C5	120.57 (12)	C15—C14—H14	120.00
C6—C5—N4	121.74 (12)	C10—C15—H15	120.00
C6—C5—C18	122.78 (12)	C14—C15—H15	120.00
N4—C5—C18	115.43 (11)	C12—C16—H16A	109.00
C5—C6—C7	117.99 (12)	C12—C16—H16B	109.00
N8—C7—N2	110.52 (11)	C12—C16—H16C	109.00
N8—C7—C6	131.23 (12)	H16A—C16—H16B	109.00
N2—C7—C6	118.22 (12)	H16A—C16—H16C	109.00
C7—N8—N9	106.71 (10)	H16B—C16—H16C	109.00
C1—N9—N8	108.99 (11)	C3—C17—H17A	109.00
C15—C10—C11	119.64 (12)	C3—C17—H17B	109.00
C15—C10—C1	120.71 (12)	C3—C17—H17C	109.00
C11—C10—C1	119.62 (11)	H17A—C17—H17B	110.00
C12—C11—C10	121.45 (12)	H17A—C17—H17C	109.00
C11—C12—C13	117.93 (13)	H17B—C17—H17C	109.00
C11—C12—C16	121.36 (13)	C18—C19—H19	120.00
C13—C12—C16	120.71 (13)	C20—C19—H19	120.00
C14—C13—C12	121.05 (13)	C19—C20—H20	120.00
C13—C14—C15	120.59 (13)	C21—C20—H20	120.00
C14—C15—C10	119.32 (13)	C20—C21—H21	120.00
C19—C18—C23	118.62 (13)	C22—C21—H21	120.00
C19—C18—C5	120.26 (12)	C21—C22—H22	120.00
C23—C18—C5	121.10 (13)	C23—C22—H22	120.00
C20—C19—C18	120.64 (14)	C18—C23—H23	120.00
C21—C20—C19	120.20 (16)	C22—C23—H23	120.00
C20—C21—C22	119.68 (15)		
			
N9—C1—N2—C7	1.13 (13)	N2—C1—C10—C15	−115.37 (15)
C10—C1—N2—C7	−172.48 (12)	N9—C1—C10—C11	−105.68 (15)
N9—C1—N2—C3	−170.93 (13)	N2—C1—C10—C11	67.00 (17)
C10—C1—N2—C3	15.5 (2)	C15—C10—C11—C12	0.7 (2)
C7—N2—C3—N4	8.25 (18)	C1—C10—C11—C12	178.35 (12)
C1—N2—C3—N4	179.34 (13)	C10—C11—C12—C13	0.3 (2)
C7—N2—C3—C17	−169.90 (12)	C10—C11—C12—C16	−179.23 (13)
C1—N2—C3—C17	1.2 (2)	C11—C12—C13—C14	−0.8 (2)
N2—C3—N4—C5	−0.57 (19)	C16—C12—C13—C14	178.73 (15)
C17—C3—N4—C5	177.53 (12)	C12—C13—C14—C15	0.3 (2)
C3—N4—C5—C6	−5.2 (2)	C13—C14—C15—C10	0.7 (2)
C3—N4—C5—C18	177.12 (12)	C11—C10—C15—C14	−1.2 (2)
N4—C5—C6—C7	3.13 (19)	C1—C10—C15—C14	−178.84 (13)
C18—C5—C6—C7	−179.37 (11)	C6—C5—C18—C19	−165.26 (14)
C1—N2—C7—N8	−1.59 (14)	N4—C5—C18—C19	12.39 (19)
C3—N2—C7—N8	171.93 (11)	C6—C5—C18—C23	13.4 (2)
C1—N2—C7—C6	176.47 (11)	N4—C5—C18—C23	−168.96 (12)
C3—N2—C7—C6	−10.01 (17)	C23—C18—C19—C20	−1.1 (2)
C5—C6—C7—N8	−178.04 (13)	C5—C18—C19—C20	177.61 (15)
C5—C6—C7—N2	4.38 (18)	C18—C19—C20—C21	0.7 (3)
N2—C7—N8—N9	1.43 (14)	C19—C20—C21—C22	0.1 (3)
C6—C7—N8—N9	−176.29 (13)	C20—C21—C22—C23	−0.5 (3)
N2—C1—N9—N8	−0.31 (14)	C21—C22—C23—C18	0.1 (2)
C10—C1—N9—N8	173.38 (11)	C19—C18—C23—C22	0.7 (2)
C7—N8—N9—C1	−0.69 (14)	C5—C18—C23—C22	−178.02 (13)
N9—C1—C10—C15	71.96 (18)		

### Hydrogen-bond geometry (Å, °)

**Table:**

*D*—H···*A*	*D*—H	H···*A*	*D*···*A*	*D*—H···*A*
C6—H6···N8^i^	0.95	2.53	3.4597 (17)	166
C23—H23···N8^i^	0.95	2.61	3.5374 (19)	167

Symmetry codes: (i) −*x*, −*y*, −*z*+1.
